# Magnetic Biochar Derived from Waste Bamboo as a Peroxymonosulfate Activator for Tetracycline Hydrochloride Degradation

**DOI:** 10.3390/molecules30112283

**Published:** 2025-05-23

**Authors:** Xingyan Huang, Yuanlong Chen, Yujia Zhang, Hongpeng Li, Shihao Xu, Xinhong Fu, Anjiu Zhao, Xiaobo Huang, Jiaming Lai

**Affiliations:** 1 College of Forestry, Sichuan Agricultural University, Chengdu 611130, China; hxy@sicau.edu.cn (X.H.); 2022304110@stu.sicau.edu.cn (Y.C.); 15608031415@163.com (S.X.); 15969700779@163.com (X.F.); anjiu_zhao@sicau.edu.cn (A.Z.); 2 Wood Industry and Furniture Engineering Key Laboratory of Sichuan Provincial Department of Education, Sichuan Agricultural University, Chengdu 611130, China; z28184747@163.com; 3 National Forestry and Grassland Administration Key Laboratory of Forest Resources Conservation and Ecological Safety on the Upper Reaches of the Yangtze River, Sichuan Agricultural University, Chengdu 611130, China; 4 Forestry Ecological Engineering in the Upper Reaches of the Yangtze River Key Laboratory of Sichuan Province, Sichuan Agricultural University, Chengdu 611130, China; 5 Research Institute of Characteristic Flowers and Trees, Chengdu Agricultural College, Chengdu 611130, China; 19181707723@163.com

**Keywords:** biochar, magnetic, peroxymonosulfate, tetracycline hydrochloride, microwave

## Abstract

Magnetic Fe and N-doped biochar (FeN-BC) was synthesized from waste bamboo through microwave pyrolysis and used as a catalyst for the degradation of tetracycline hydrochloride (TC) with peroxymonosulfate (PMS). The results showed that doping with Fe improved the recovery performance of biochar and the N-doping enhanced the activity of PMS. Simultaneously, it achieved a high degradation efficiency for TC (93%) under optimized conditions within 30 min. Electron paramagnetic resonance (EPR) and quenching experiments indicated that the main active radicals present in the experiment were SO_4_^•−^ and •OH. Additionally, FeN-BC demonstrated good catalytic performance in the TC degradation process in a real water environment after five cycles. This work presents a practical strategy for preparing magnetic biochar to degrade organic pollutants from wastewater.

## 1. Introduction

With the continuous development of the global medical industry, the widespread use of antibiotics has played an important role in safeguarding human health and promoting the development of animal husbandry. However, the environmental problems caused by their excessive discharge are becoming increasingly serious. According to statistics, the global annual use of antibiotics exceeds 100,000 tons, of which about 30–90% enter the water and soil environment in the form of prototypes or metabolites [1]. As a typical representative of tetracycline antibiotics, tetracycline hydrochloride (TC) is widely used in medical treatment, animal husbandry, and aquaculture due to its broad-spectrum antibacterial properties, low cost, and high stability [2]. However, the persistence of TC in the environment poses multiple risks, and the accumulation of TC in the environment accelerates the lateral transmission of drug-resistance genes, threatens the stability of microbial communities, and even endangers human health through food chain [3,4]. According to the works about the distributions of antibiotics in China, the maximum concentration of TC in surface water could exceed 5 µg/L [5].

As a new advanced oxidation process (AOP), peroxymonosulfate (PMS) has been widely used in the treatment of refractory organic pollutants in recent years due to its strong oxidation ability, diverse reaction pathways, and high environmental friendliness [6,7]. Compared to traditional persulfate, the asymmetric structure of PMS (HO-O-SO_3_^−^) makes it more susceptible to being activated, generating sulfate radicals (SO_4_^•−^, oxidation potential 2.5–3.1 V) and hydroxyl radicals (•OH, oxidation potential 1.8–2.7 V) by breaking O-O bonds. At the same time, selective oxidation can be achieved through non-free radical pathways [8]. The results show that the metal activity checkpoint directly triggers the free radical chain reaction through the valence cycle, and the defective structure or oxygen-containing functional groups in the carbon matrix can promote the adsorption and surface electron transfer of PMS, formation of non-radical-dominated oxidation pathways [9,10].

Carbon materials have attracted increasing attention as catalysts due to their large specific surface area, good biocompatibility, good stability, and high electrical conductivity. Some carbon-based materials, such as carbon nanotubes [11], activated carbon [12], and reduced graphene oxide [13], have been proven to have an effective persulfate activation effect due to the presence of catalytic centers (defects and oxygen-containing functional groups) in the sp^2^-hybridized carbon matrix [14]. However, the biochar obtained by direct pyrolysis of biomass has limited surface functional groups and low specific surface area, and cannot be used as a high-performance carbon material [15]. Therefore, preparing biochar with abundant active functional groups and developed pore structure is crucial for the high-value-added utilization of biomass. In recent years, N-doping has become a feasible strategy to enhance the physicochemical properties of biochar. N-doped biochar has abundant nitrogen-containing functional groups, such as pyrrole-N, pyridine-N, and graphite N. The reason for the formation of graphite N is that N atoms are transferred to the carbon skeleton and replaced C atoms in the graphite structure [16,17]. Graphite N has a higher electronegativity, which is conducive to more electrons transferring to the N atom and reducing the overall charge density in the carbon matrix, thereby improving the π-π interaction [18,19]. Nitrogen-containing groups, such as pyrrole-N, pyridine-N, and N-O, provide more absorption active sites on the surface of biochar, improve the pore structure of the absorbent, and enhance the absorption performance by generating a more stable combination with antibiotics [20,21]. Separating the used biochar from the liquid phase is a complex and time-consuming process. The Fe and N co-doping strategy not only realizes fast and simple solid-liquid separation but also maintains the efficient absorption performance of antibiotics [22,23]. In addition, the biochar loaded with Fe-N decomposes at high temperatures to form more graphite N, which can adjust the electronic structure of the carbon matrix and improve the surface coordination and π-π stacking between biochar and antibiotics [24,25]. The carbon materials are not easily recyclable after the catalytic degradation process. Magnetic biochar was developed to solve the problem of secondary pollution. As a functional environmental remediation material, magnetic biochar has shown significant potential in the field of refractory organic pollutant treatment in recent years [26]. Combining magnetic metal materials with biochar matrix has the characteristics of adsorption and enrichment, catalytic activity, and magnetic separation recovery capabilities [27,28,29], especially suitable for continuous flow wastewater treatment scenarios [30].

In this research, waste bamboo was used as a biomass substrate, and iron metal and nitrogen sources were doped into biochar. Magnetic Fe and N-doped biochar (FeN-BC) was prepared by simple and effective one-step microwave pyrolysis. Tetracycline hydrochloride, which is widely distributed in the water environment, was selected as a representative pollutant for the degradation process. The Fe additional ratio was optimized through the characterization experiments and catalytic experiments of magnetic biochar. The influences of catalyst amount, PMS dosage, temperature, and other conditions on the catalytic degradation process were analyzed, and the catalytic mechanism of the FeN-BC/PMS system was studied.

## 2. Results and Discussion

### 2.1. Characterizations

**Figure 1:**
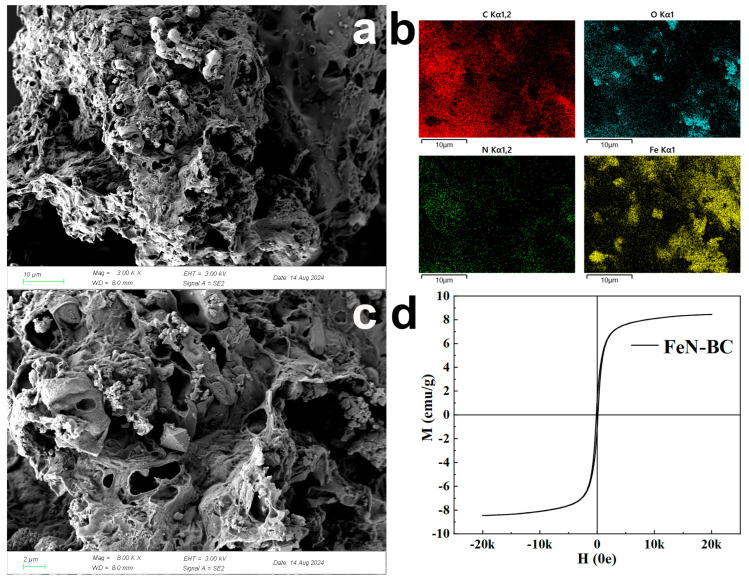
(**a**) SEM 10 μm image of FeN-BC, (**b**) EDS elements distribution of FeN-BC, (**c**) SEM 2 μm image of FeN-BC, (**d**) hysteresis loop.

The morphology and microstructure of the biochar were characterized by SEM. As shown in Figure 1a,c there was a pore structure on the surface of the FeN-BC, and the surface was relatively rough, which was related to the carbonization temperature and time of the material according to our previous work [31]. At the same time, relatively regular Fe_2_O_3_ particles were observed on the surface of the biochar, and some of them showed a cluster shape. The successful introduction of Fe was also proved by EDS (Figure 1b) analysis, indicating that Fe had been incorporated into the biochar surface. The dispersed Fe_2_O_3_ particles introduced magnetism to the FeN-BC. The magnetism of FeN-BC was evaluated by a hysteresis curve recorded at room temperature (Figure 1d). The values of magnetic and residual magnetic forces are negligible, indicating that FeN-BC exhibited typical superparamagnetic behavior. The magnetic attraction and remanence forces are very small and mainly caused by a small fraction of larger iron oxide nanoparticles [32]. The saturation magnetic value of FeN-BC is 8.44 emu/g.

**Figure 2:**
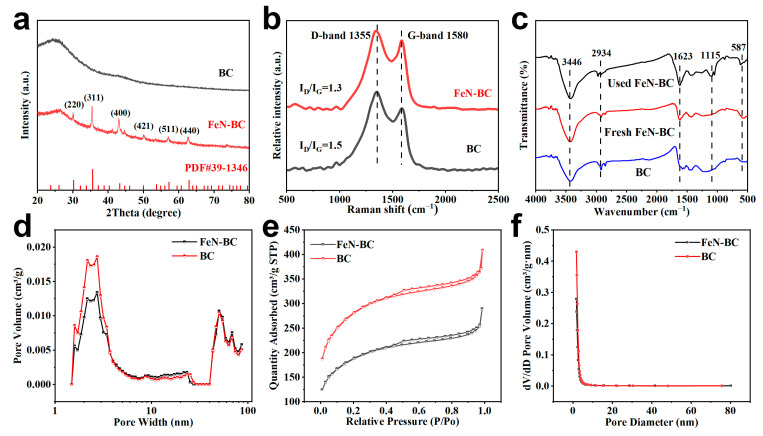
(**a**) XRD profiles of BC and FeN-BC, (**b**) Raman profiles of BC and FeN-BC, (**c**) FTIR test profiles of BC and FeN-BC using before-and-after comparison, (**d**–**f**) BET test profiles of BC and FeN-BC.

The crystal structure of FeN-BC was characterized by an X-ray diffractometer shown in Figure 2a. The catalyst contained Fe, had a wide diffraction peak at about 25°, which was located on the (002) plane of the graphitized structure in biochar [33]. At the same time, no obvious peaks were observed in the X-ray diffraction pattern of BC, indicating the existence of an amorphous carbon structure [34]. The diffraction peaks of 30.24°, 35.63°, 43.28°, 50.00°, 57.27°, and 62.92° were indexed to the (220), (311), (400), (421), (511), and (440) surfaces of Fe_2_O_3_, respectively. The stronger and sharper diffraction peaks of Fe_2_O_3_ in FeN-BC indicated that it had a better crystalline phase [32].

Raman spectroscopy can be used to study the form of carbon atoms in materials. From Figure 2b, FeN-BC and BC showed two characteristic peaks at 1355 cm^−1^ and 1580 cm^−1^, corresponding to D and G bands, respectively. Their strength indicated the degree of graphitization and defects in carbon materials. The I_D_/I_G_ ratios of BC and FeN-BC were 1.5 and 1.3, respectively. This phenomenon is due to the fact that the doped N in the material disrupts the carbon network structure, resulting in an increase in material defects [35]. It was shown that the defects of FeN-BC were relatively reduced. Therefore, the structure of the FeN-BC could be more ordered with a uniform and high charge transport efficiency electric structure, which facilitated electron transfer process [36].

Based on FTIR analysis in Figure 2c, the broad peak of FeN-BC at 3446 cm^−1^ corresponded to the stretching vibration of hydroxyl groups (O-H) on the surface of biochar or adsorbed water [37]. The presence of O-H had a positive effect on the activation of PMS [38]. Absorption peaks at 2936 cm^−1^ indicated the existence of aliphatic C-H bonds in the material, which was possibly derived from the organic structure of biomass carbonation residues. The characteristic peaks of 1623 cm^−1^ could be attributed to the C=C vibration of the aromatic ring or the C=O stretching vibration of the carboxylic acid group [39]. These functional groups could promote the activation of PMS through electron transfer. The peak at 1105 cm^−1^ suggested the existence of a C-O bond, which may be related to either phenolic hydroxyl groups, further enhancing surface reactivity. The absorption peak at 589 cm^−1^, confirmed that iron species are successfully supported on the surface of biochar [40,41].

The N_2_ absorption/desorption isotherms and pore size distribution curves for BC and FeN-BC are shown in Figure 2d–f. The N_2_ adsorption lines for BC and FeN-BC were classified as type IV adsorption lines with H_4_ type hysteresis loops (Figure 2e), demonstrating the presence of mesoporous structures of 2–50 nm (Figure 2d,f) [42]. The specific surface area of FeN-BC (662.99 m^2^/g) was less than BC (990.0 m^2^/g). This is because Fe is added to the BC surface, formin Fe_2_O_3_ agglomeration, resulting in clogging of the pore structure [43].

### 2.2. Catalytic Degradation Performance

**Figure 3:**
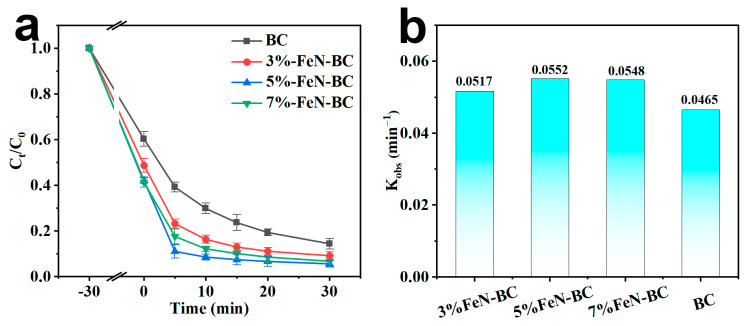
(**a**) The effect of Fe^3+^/Fe^2+^ dose on the degradation of TC and (**b**) the degradation kinetic efficiency of TC ([TC] = 50 mg/L, [catalyst] = 0.1 g/L, [PMS] = 3 mM, T = 25 °C, no pH regulation).

**Figure 4:**
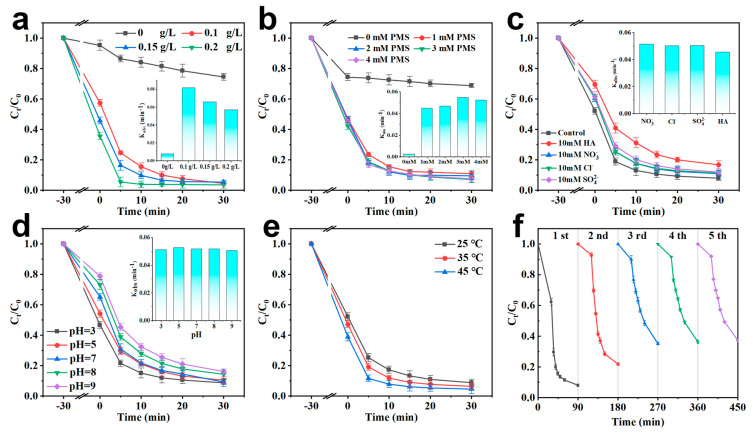
(**a**) the amount of catalyst, (**b**) the amount of PMS, (**c**) the effect of different anions on the degradation of TC, the illustration is the corresponding first-order rate constant, (**d**) the initial pH, (**e**) temperature, (**f**) the number of repeated uses of FeN-BC, [TC] = 50 mg/L, [catalyst] = 0.1 g/L (except a), [PMS] = 3 mM (except b), T = 25 °C (except for e), without pH adjustment (except for d)).

The effect of iron content in the FeN-BC on the catalytic conditions was evaluated. Different proportions of metallic iron were added to the FeN-BC, respectively. At the same time, biochar without iron was prepared for catalytic comparison, and the optimal conditions were evaluated. The TC removal efficiency of four catalysts was carried out in the presence of PMS, as shown in Figure 3. Here, TC degradation follows pseudo-first-order kinetics, which was described by kinetic equations: −ln (C_t_/C_0_) = K_obs_t, where K_obs_ is the apparent rate constant, C_t_ and C_0_ are TC concentrations at time t and initial, respectively. As shown in Figure 3, 5% FeN-BC was the most effective catalyst compared comprehensively. Its K_obs_ (0.0552 min^−1^) compared with 3% FeN-BC, the increase in catalytic efficiency, and K_obs_ of 7% FeN-BC decreased may be due to the aggregation of iron oxide particles. As shown in Figure 4a,b, when only 5% FeN-BC or PMS was added to the TC solution, the removal efficiency of TC was greatly reduced. It was suggested that the adsorption of TC on the catalyst surface and the inherent oxidation ability of PMS were negligible. In the 5% FeN-BC/PMS system, TC was degraded 90% in 10 min with the participation of PMS, indicating that 5% FeN-BC had a strong catalytic ability for PMS activation. For comparison, BC without iron was also investigated. At the same time, the degradation efficiency was 70%, indicating that the presence of iron showed a positive effect on catalysis. Although the participation of iron in the BET determination occupied a part of the BET surface area, from a catalytic point of view, it did not hinder the degradation ability. With increasing Fe^3+^/Fe^2+^ dose in different magnetic Fe and N-doped biochar samples, the initial TC adsorption performance increased from 39.77% to 51.27%, 58.00%, and 58.69%, respectively. One possible reason for this phenomenon could be the larger average of FeN-BC (2.70 nm) than BC (2.56 nm) (Table S1). It might facilitate TC adsorption before the degradation process. The other possible reason to influence TC adsorption capacity might be electrostatic attraction and functional groups. The surficial Fe of FeN-BC could facilitate surface reaction and ion exchange processes [44].

The effects of catalyst and PMS dosage, solution pH, and reaction temperature on TC degradation in the FeN-BC/PMS system were investigated. As shown in Figure 4a, when the catalyst dose was increased from 0.1 g/L to 0.2 g/L, K_obs_ decreased from 0.0815 min^−1^ to 0.057 min^−1^. Combined with the degradation efficiency and reaction rate, it was demonstrated that the higher catalyst dose had a positive impact on the TC removal efficiency due to the more active points. At 30 min, the critical point had been reached. Considering the maximum utilization of the catalyst and cost control, 0.1 g/L was chosen as the optimal dose. The results shown in Figure 4b indicated that with the increase in the dose of PMS, the TC degradation rate increased gradually until the maximum utilization was achieved. When the PMS dose was increased to 3 mM, the degradation efficiency of 93% could be attained in 30 min. However, when the PMS concentration reached 4 mM, the degradation efficiency was observed to decrease. This could be attributed to sulfate quenching caused by excessive addition of PMS [45].

In the range of solution pH = 3–9, the degradation efficiency of TC by the FeN-BC/PMS system was investigated. As shown in Figure 4d, in the range of pH = 3–7, the removal efficiency of TC was more than 90%, indicating that the catalyst system had a wide working pH range. When the solution pH was 11, the degradation rate was decreased to 84%, which was due to the self-decomposition of PMS into sulfate ions (SO_4_^2−^) caused by the reduction of free radicals [46]. Compared with the traditional Fenton reaction system, the FeN-BC/PMS system had a good catalytic performance in a wide pH range. In addition, the release concentration of Fe in the solution after oxidation of the FeN-BC/PMS system under normal pH conditions was also studied. After one use, when the solution pH was 4.6, the leaching amount of Fe in the solution was 1 mg/L, which was much smaller than the reported pH = 3.0 (4.51 mg/L) and pH = 5.0 (3.83 mg/L) [47]. It was shown that FeN-BC had strong stability. In the selected experimental pH range, considering the corresponding TC removal effect, the optimal operating pH range of the FeN-BC/PMS system was 3–7.

Figure 4e shows the effect of reaction temperature on the degradation of TC in the FeN-BC/PMS system. When the temperature increased, the removal rate of TC increased synchronously to 97%. The reaction rates were 0.0559 min^−1^ (25 °C), 0.0619 min^−1^ (35 °C), and 0.0627 min^−1^ (45 °C), respectively. This was because the increased temperature could provide more energy for the reactant molecules to accelerate the reaction speed. Thermal activation prompts PMS to convert into active radicals at higher temperatures [48]. More detailed experiments and calculations about thermodynamic data will be conducted in the future work. The results of the total organic carbon (TOC) analysis showed that after 30 min of adsorption and 30 min of catalytic degradation, the TOC removal rate reached 48.1% (Figure S1), indicating that half of the total carbon (TC) could be degraded into H_2_O and CO_2_.

In addition, the stability and reusability of FeN-BC were also evaluated. As shown in Figure 4f, the removal efficiency of TC decreased gradually with increasing cycles. 63% of TC could be removed within 90 min after five cycles, suggesting that the magnetic biochar had a high reusability and stability. The deactivation of catalytic performance was related to the coverage effect of degradation intermediates on the surface of FeN-BC or the inevitable consumption of active sites [49]. In addition, the degradation efficiencies of the FeN-BC/(PMS) system for other antibiotics, namely doxycycline (DC), oxytetracycline (OTC), and chlortetracycline (CTC), were 86.2%, 84.1%, and 77.9%, respectively (Figure S2). This result indicated that the FeN-BC/PMS system had a wide range of applicability and could effectively remediate antibiotics in wastewater.

Due to the prevalence of common anions in wastewater, the performance of catalysts could be affected in a variety of ways [50]. Therefore, the effects of Cl^−^, NO_3_^−^, and SO_4_^2−^ on the degradation process were studied. In Figure 4c, it could be observed that the presence of Cl^−^, NO_3_^−^, and SO_4_^2−^ had a small inhibitory effect on TC removal. The pseudo-first-order kinetic constants (K_obs_) of TC degradation in the presence of Cl^−^, NO_3_^−^, and SO_4_^2−^ were calculated to be 0.0503, 0.0515, and 0.0505 min^−1^, respectively. According to previous studies, SO_4_^•−^ and •OH can both react with Cl^−^ and NO_3_^−^ to produce •CI/•CI_2_^−^, NO_3_^−^ with relatively low oxidation potential, resulting in deterioration of degradation performance [45,51,52]. Humic acid (HA) was used as the representative to evaluate the resistance of FeN-BC to natural organic matter. At 10 mM, the pseudo-first-order kinetic constants (K_obs_) of TC degradation were 0.0456 min^−1^. Although the degradation rate of TC decreased in the presence of HA, the removal rate of TC was still 84%. The rich hydroxyl groups in HA may have a slight adverse effect on the FeN-BC/PMS system because these groups can extinguish free radicals and block the active point on the catalyst surface [50,53]. The good resistance of FeN-BC to anions and HA confirmed its excellent catalytic activity and suggested its potential application in practical organic wastewater purification.

### 2.3. Degradation Mechanism

**Figure 5:**
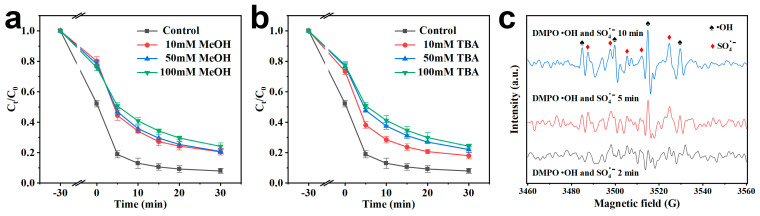
(**a**,**b**) Effect of free radical scavenging on the degradation of TC in FeN-BC/PMS system, (**c**) Radical capture spectra of EPR, ([TC] = 50 mg/L, [catalyst] = 0.1 g/L, [PMS] = 3 mM, T= 25 °C, without pH regulation).

In previous studies, SO_4_^•−^ and •OH were the main reactive oxygen species presented in PMS-based AOP [54]. Selection of methanol as a quencher for SO_4_^•−^ and •OH (K SO_4_^•−^ = 1.6–7.7 × 10^7^ M^−1^S^−1^); (K•OH =1.2–2.8 × 10^9^ M^−1^S^−1^) [55]. TBA is only valid for •OH (3.8–7.6 × 10^8^ M^−1^S^−1^) quenching [52]. As shown in Figure 5, the catalytic effect was suppressed in the reaction due to the consumption of SO_4_^•−^ and •OH by scavengers. After the addition of methanol to the reaction system, the degradation rate and removal rate of TC were reduced to 80% and 0.0424 min^−1^, respectively. In addition, the degradation rate and removal rate of TC decreased to 82% and 0.0431 min^−1^, respectively, after the addition of TBA, indicating that the degradation of TC was not only caused by •OH. Characteristic radical signals involving DMPO- •OH and DMPO- SO_4_^•−^ appeared in electron paramagnetic resonance (EPR) spectra (Figure 5c), indicating that •OH and SO_4_^•−^ were the main radicals in the FeN-BC/PMS system [56].

**Figure 6:**
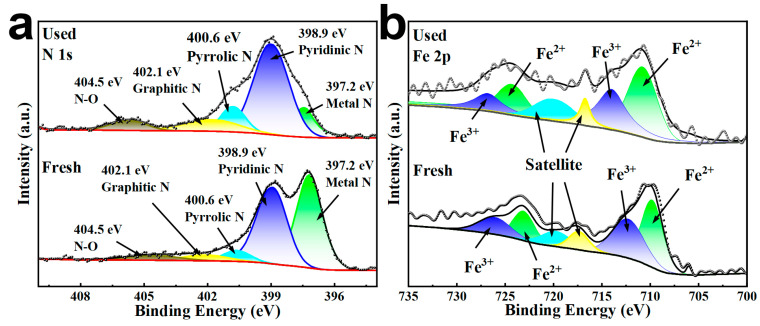
(**a**) N 1s, (**b**) Fe 2p before and after FeN-BC reaction.

X-ray photoelectron spectroscopy (XPS) showed the presence of Fe and N on the surface of FeN-BC as shown in Figure 6. The peaks of 709.8 and 723.1 eV and the peaks of 712.2 and 726.0 eV confirmed the presence of Fe^2+^ and Fe^3+^ in the FeN-BC, respectively, indicating that the magnetic iron particles were loaded on the surface of the biochar [57]. The N 1s spectra of FeN-BC are shown in the figure, and the five matched peaks correspond to metal nitride (297.2 eV), pyridine N (398.9 eV), pyrrole N (400.6 eV), graphite N (402.1 eV), and nitrogen oxide (404.5 eV). Among them, pyridine N in FeN-BC could improve the production of SO_4_^•−^ and •OH radicals as the Lewis basic site during AOPs [35]. At the same time, it could be observed that the content of metal nitride decreases after use, so it could be known that metal nitride was consumed in the reaction. The N atom in the metal nitride imparts a positive charge to the neighboring C atom [58]. Enhanced absorption of PMS molecules at C atoms associated with the metal nitride point has been reported [59]. Therefore, N atoms in metal nitrides act as electron receptors, promoting PMS depletion. These findings suggested that N incorporation played a crucial role in enhancing PMS activation processes and promoting electron transfer.

## 3. Experimental

### 3.1. Materials

Three-year-old raw Bambusa rigida was collected from Ya’an City, Sichuan Province. Urea (CH_4_N_2_O), potassium hydroxide (KOH), Sodium chloride (NaCl), sodium nitrate (NaNO_3_), sulfate anhydrous (Na_2_SO_4_) tetracycline hydrochloride (TC), methanol (MeOH), ferric chloride-hexahydrate (FeCl_3_·6H_2_O), ferrous chloride-tetrahydrate (FeCl_2_·4H_2_O), peroxide peroxymonosulfate (PMS), sodium thiosulfate (Na_2_S_2_O_3_), and tert-butanol (TBA) were all purchased from Chengdu Haoboyou Co., Ltd. (Chengdu, China).

### 3.2. Preparation of FeN-BC

The bamboo was ground and screened to 50–60 mesh and then dried in the oven at 105 °C. 6 g of bamboo powder, 6 g of KOH,10 g of urea, 0.184 g of FeCl_2_, and 0.25 g of FeCl_3_ were mixed with 80 mL of deionized water, and then the mixed solution was stirred at room temperature for 8 h. Then the mixed solution was placed in an oven at 80 °C for 8 h. A 10 g sample was poured into a ceramic crucible, and then the crucible was placed into a microwave reactor. Nitrogen was introduced to create an inert atmosphere. After the reactor was filled with nitrogen, microwave heating was carried out for 10 min to produce FeN-BC (5%-FeN-BC). Then, the FeN-BC was washed to neutral and dried in an oven at 60 °C overnight. Magnetic biochar with different Fe^3+^/Fe^2+^ contents (3%-FeN-BC and 7%-FeN-BC) and biochar without Fe^3+^/Fe^2+^ (BC) were prepared by the same method.

### 3.3. Characterization of FeN-BC

The crystal structure of the samples was analyzed by X-ray diffraction spectrometer (XRD, Panalytical Aeris type, Malvern, UK) on an X-ray diffractometer at Co Kα (λ = 1.78897 Å). Raman spectra were obtained by a Raman spectrophotometer (Raman, Horiba LabRAM HR Evolution, Kyoto, Japan). The catalysts were characterized by field emission scanning electron microscopy (SEM, ZEISS GeminiSEM 300, Jena, Germany) with an energy dispersive spectroscope (EDS). N_2_ adsorption-desorption isotherms were determined by Brunauer–Emmett–Teller (BET, Micromeritics ASAP 2460, Norcross, GA, USA), and the specific surface area and pore structure of the resulting materials were investigated. The surface chemistry and element composition were analyzed by X-ray photoelectron spectroscopy (XPS, Thermo Scientific ESCALAB Xi+, Waltham, MA, USA) instrument. The magnetic properties of the samples were measured by vibrating sample magnetometer (VSM, Lakeshore 8604, Carson, CA, USA) at room temperature, the electron paramagnetic resonance spectrometer (EPR, Bruker EMXplus-6/1, Billerica, MA, USA) test for active species detection was carried out with 5,5-dimethyl-1-pyrroline N-oxide (DMPO) and 2,2,6,6-tetramethyl-piperidinol (TEMP) as spin trapping agents, the catalytic effect was determined by UV-Vis spectrophotometer (UV-Vis, UV-1900i, Shimadu, Suzhou, China), the functional groups and molecular structures of the samples were determined by fourier-transform infrared spectroscopy (FTIR, Thermo Fisher Scientific Nicolet iS20, Waltham, MA, USA), and the total organic carbon content in the reaction solution was determined by elementar vario total organic carbon analyzer (TOC, Shimadu TOC-L CPH, Kyoto, Japan).

### 3.4. Catalytic Degradation of TC

10 mg of FeN-BC was poured into 100 mL of TC solution (50 mg/L), and adsorption-desorption equilibrium was performed with magnetic stirring for 30 min. After that, 3 mM of peroxymonosulfate solution (PMS) was added to the TC solution for catalytic degradation. After a fixed time interval of 5 min, 3 mL of the reaction solution sample was removed and quenched with 20 μL of Na_2_S_2_O_3_ (0.1 M). The solid particles were filtered through a 0.22 μm filtration membrane, and the remaining amount of TC was measured by UV-Vis spectrophotometer. To determine the optimal catalytic degradation conditions, different catalyst dosages (0–0.2 g/L), PMS concentrations (0–4 mM), organic matter and anions (HA, NO_3_^−^, Cl^−^, SO_4_^2−^), TC solutions pH (3–9), and TC solution temperatures (25–45 °C) were investigated.

## 4. Conclusions

In this study, magnetic biochar was prepared by a simple microwave pyrolysis method using waste bamboo as biomass and Fe, N as dopant. Compared with biochar without iron doping, the doping of iron solved the problem of the separation and recycling of biochar after degradation and enhanced the catalytic activity of PMS. Without adjusting the pH, 93% of TC could be removed in the FeN-BC/PMS system within 60 min. FeN-BC/PMS mainly produced SO_4_^•−^ and •OH for the TC degradation process. The FeN-BC had a wide range of pH effectiveness, high stability, and good reusability, which was conducive to practical application. It was found that iron oxides and nitrogen doping are both catalytic active points, and the free radical oxidation pathways played a leading role in TC degradation. This work provides a practical strategy for the preparation of magnetic biochar for TC degradation.

## Data Availability

All data in this study are included in the manuscript.

## Supplementary Materials

The following supporting information can be downloaded at: https://www.mdpi.com/article/10.3390/molecules30112283/s1. Figure S1: The removal efficiency of TOC in TC degradation.; Figure S2: The degradation efficiencies of TCs.; Table S1: Porous characterization of the FeN-BC and BC.
