# Supplementary material for: Magnetic Biochar Derived from Waste Bamboo as a Peroxymonosulfate Activator for Tetracycline Hydrochloride Degradation
Source: Molecules. 2025 May 23;30(11):2283. doi: 10.3390/molecules30112283 (PMC12156303; doi:10.3390/molecules30112283)
Supplement: Supplementary file 1 [file molecules-30-02283-s001.zip › molecules-3611690-supplementary.pdf]

Magnetic biochar derived from waste bamboo as a peroxymonosulfate activator for tetracycline hydrochloride degradation

Xingyan Huang [a, b, c, 1], Yuanlong Chen [a, b, c, 1], Yujia Zhang [b, c], Hongpeng Li [d], Shihao Xu [a, b, c], Xinhong Fu [a, b, c], Anjiu Zhao [a, b, c], Xiaobo Huang [a, b, c, *], Jiaming Lai [a, b, c, *]

[a] *College of Forestry Sichuan Agricultural University Chengdu Sichuan 611130 China*

[b] *Wood Industry and Furniture Engineering Key Laboratory of Sichuan Provincial Department of Education 611130 Chengdu Sichuan P.R. China*

[c] *National Forestry and Grassland Administration Key Laboratory of Forest Resources Conservation and Ecological Safety on the Upper Reaches of the Yangtze River & Forestry Ecological Engineering in the Upper Reaches of the Yangtze River Key Laboratory of Sichuan Province Chengdu 611130 China*

[d] *Research Institute of Characteristic Flowers and Trees Chengdu Agricultural College Chengdu Sichuan 611130 China*

[1]Co-first authors: Xingyan Huang, and Yuanlong Chen.

*Corresponding authors: Xiaobo Huang, berber@stu.sicau.edu.cn; Jiaming Lai, ljm4936@yahoo.com.cn.

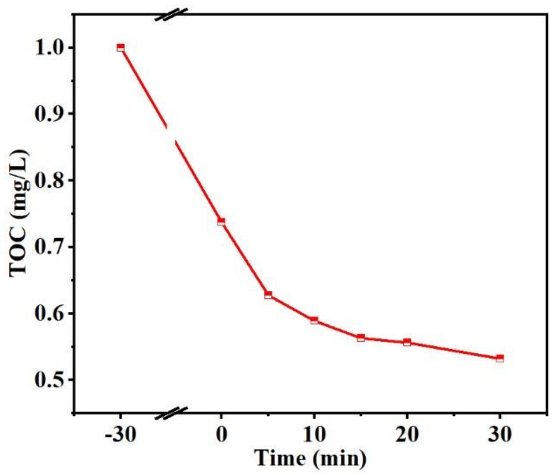

Figure S1. The removal efficiency of TOC in TC degradation.

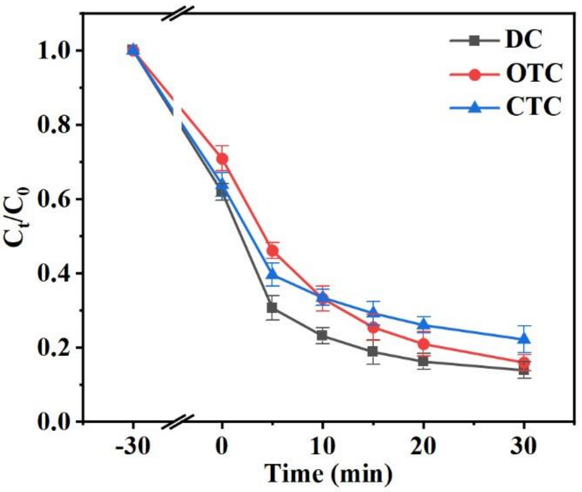

Figure S2. the degradation efficiencies of TCs. (The default experiment conditions: FeN-BC = 0.1 g/L, PMS = 3 mM, TC = DC = OTC =CTC = 50 mg/L, T = 25 °C no pH regulation)

Table S1. Porous characterization of the FeN-BC and BC.

	FeN-BC	BC
BET surface area (m^2 g^{-1})	662.99	990.00
Total pore volume (cm^3 g^{-1})	0.448	0.632
Average pore size (nm)	2.70	2.56
